# Structural Analysis of Triacylglycerols by Using a MALDI-TOF/TOF System with Monoisotopic Precursor Selection

**DOI:** 10.1007/s13361-012-0513-9

**Published:** 2012-12-18

**Authors:** Ayumi Kubo, Takaya Satoh, Yoshiyuki Itoh, Masahiro Hashimoto, Jun Tamura, Robert B. Cody

**Affiliations:** 1 JEOL Ltd., Tokyo, Japan; 2 JEOL USA, Inc., Peabody, MA 01960 USA

**Keywords:** MALDI, TOF/TOF, Charge-remote fragmentation, High-energy CID, Triacylglycerols, MS/MS, Lipids, Collision-induced dissociation

## Abstract

A new MALDI-TOF/TOF system with monoisotopic precursor selection was applied to the analysis of triacylglycerols in an olive oil sample. Monoisotopic precursor selection made it possible to obtain product-ion mass spectra without interference from species that differed by a single double bond. Complete structure determination of all triacylglycerols, including structural isomers, was made possible by interpreting the charge-remote fragmentation resulting from high-energy collision-induced dissociation (CID) of the sodiated triacylglycerols.

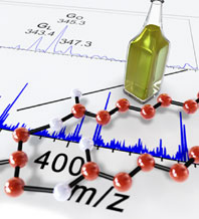

## Introduction

Triacylglycerols (TAGs or triglycerides) are comprised of three fatty acids esterified with glycerol. Because TAGs are the major components in animal fats and vegetable oils, the analysis of TAGs is biologically important and crucial for quality control of food products.

Recent mass spectrometric approaches to the analysis of TAGs have made use of atmospheric pressure chemical ionization (APCI) [1–5] or electrospray ionization (ESI) [6–10] and tandem mass spectrometry (MS/MS).

Using ESI and a triple quadrupole mass spectrometer, Hsu and Turk [6] reported that collisional activation of lithium adducts of TAGs can provide structural information about the acyl groups. Low-energy collision-induced dissociation (CID) of cationized TAGs does not provide information about the position of the double bonds. However, the CID fragments of unusual dilithiated species was shown to be dependent on double bond location. Byrdwell and Neff [7] reported a method based on dual parallel ESI and APCI combined with tandem mass spectrometry for the analysis of TAGs and their oxidation products. McAnoy et al. [8] used ESI with a linear ion trap to characterize TAG components within a complex mixture of neutral lipids from cell extracts.

High-energy CID is an especially attractive approach for TAG analysis because charge-remote fragmentation [11–22] provides a great deal of information about lipid structure. The complete structural characterization of TAGs was reported in 1998 by Cheng et al. using fast atom bombardment (FAB) and tandem magnetic sector mass spectrometry with high-energy CID fragmentation of the [M + Na]^+^ species [21]. All TAG structural features could be determined except stereochemistry.

However, large tandem magnetic sector mass spectrometers have fallen out of favor in recent years and high-energy CID appeared destined to become a “lost art” until the introduction of tandem time-of-flight (TOF/TOF) mass spectrometers by Cotter and Cornish in 1993 [22]. Recently, Pittenauer and Allmaier showed that TOF/TOF mass spectrometers have the potential to provide the same complete structural information as a tandem magnetic sector mass spectrometer [23]. The principal limitation of this method was found to be the poor MS-I selectivity (a 4 to 6 u window) of the TOF/TOF system, making it impractical to select precursor ions for TAGs with compositions that differ by two hydrogens. The authors concluded that a LC/MALDI-MS/MS approach might be required to make use of charge-remote fragmentations to characterize TAGs in complex mixtures.

We have developed a tandem time-of-flight mass spectrometer featuring high precursor ion selectivity that resolves the problem of poor MS-I selectivity [24]. The mass spectrometer uses multi-turn and “perfect focusing” ion optics [25] to fit a very long (17-m) flight path into a compact space [26]. In TOF/TOF mode, an ion gate positioned at the 15 m point in the spiral ion flight path is used to isolate and guide the precursor ion into a gas-filled collision chamber. The long flight path provides ample time separation prior to precursor ion selection, resulting in unit precursor selectivity. The precursor ions undergo 20 kV collisions with a target gas and are subjected to a 9 kV post-acceleration into an offset parabolic reflectron with wide energy acceptance.

Monoisotopic precursor selection combined with high-energy CID is the key to using TOF/TOF for structural analysis of triacylglycerols in complex mixtures. This paper describes the method for structural analysis with this system and reports the complete structural analysis of TAGs, including isomers, in a commercial olive oil sample.

## Experimental

### Materials and Chemicals

A triacylglycerol standard (1-palmitoyl-2-oleoyl-3-linoleoyl-rac-glycerol), matrix (2,5-dihydroxybenzoic acid or DHB), and cationizing agent (sodium trifluoroacetate), were purchased from Sigma-Aldrich (St. Louis, MO). A trioleoylglycerol (triolein) standard was purchased from TCI. Tetrahydrofuran (THF) was purchased from Wako (Osaka, Japan) and olive oil was purchased from local stores. The triacylglycerol standard, including triolein, and the olive oil were dissolved in THF at respective concentrations of 100 pmol/uL and 10 ug/uL. A solution of sodium trifluoroacetate and DHB was dissolved in THF at respective concentrations of 1 ug/uL and 20 ug/uL, and added to the samples at a volume ratio of 1:1:2. The resulting mixture was loaded onto an MTP 96-hole hairline plate (JEOL Ltd., Akishima Japan) at a volume of 1 uL per spot.

### MALDI Mass Spectrometry

A JMS-S3000 Spiral TOF (JEOL Ltd., Akishima, Japan) equipped with the TOF/TOF option was used for all measurements. The laser was a Nd-YLF laser operated at a wavelength of 349 nm. The laser intensity and the detector voltage were set to prevent triacylglycerol peaks from saturating. The extraction delay was optimized to 400 ns to provide a resolving power (FWHM) of approximately 50,000 for the TAG peaks in MS-I mode. For product-ion mass spectrum acquisition, helium collision gas was introduced to attenuate the precursor ion abundance to approximately 50 % of the initial value. The laser was operated at a repetition rate of 1000 Hz. Spectra were acquired at a rate of two spectra per s and 500 spectra were accumulated for each product-ion mass spectrum shown here.

## Results and Discussion

**Figure 1:**
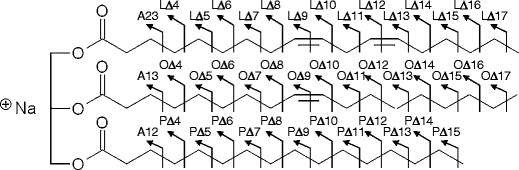
Structure and charge-remote fragmentation of sodiated 1-palmitoyl-2-oleoyl-3-linoleoyl-rac-glycerol

Figure 1 shows the structure of 1-palmitoyl-2-oleoyl-3-linoleoyl-rac-glycerol. The structure shows (18:1) oleic acid, (16:0) palmitic acid, and (18:2) linoleic acid substituents at position *sn*-*2* (the site that determines the stereochemistry) and positions *sn*-*1 and sn*-*3*, respectively. In this article, we have labeled the fatty acid substituents at positions *sn*-*1* and *sn*-*3* as “*sn*-*1*/*sn*-*3*.” The substituents at *sn*-*1* and *sn*-*3* are indistinguishable by mass spectrometry because the steric structure of triacylglycerol cannot be identified by mass spectrometry. Each fragmentation path is assigned as shown in Figure 1, and is labeled alphabetically. Each letter represents the initial letter of the fatty acid, and the accompanying number represents the bonding position in each fatty acid. The labeling for TAGs such as “TAG(54:3)” follows the convention where the numeral on the left in parentheses represents the total number of acyl carbon chains and the numeral on the right represents the total number of unsaturated bonds at fatty acid moieties.

**Figure 2:**
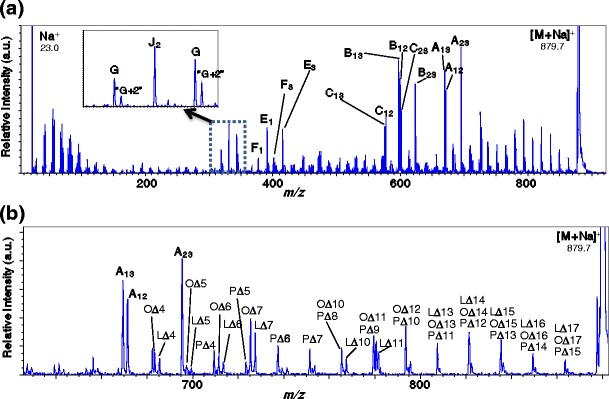
Product-ion mass spectrum for sodiated 1-palmitoyl-2-oleoyl-3-linoleoyl-rac-glycerol, (**a**) entire mass range; (**b**) *m*/*z* 650–890 magnified)

The major species observed for 1-palmitoyl-2-oleoyl-3-linoleoyl-rac-glycerol was the sodiated molecule [M + Na]^+^. Figure 2 shows the product ion spectrum acquired by selecting the monoisotopic ion of this species. The resulting fragment ions are solely monoisotopic ions as well because a monoisotopic precursor ion was selected. Thus, each fragmentation path is observed as a single peak on the product-ion mass spectrum. Figure 2a shows the entire mass range of the product-ion spectrum. The Na^+^ peak detected at *m*/*z* 23.0 confirms that the precursor ion is indeed [M + Na]^+^. Peaks characteristic of fatty acid fragmentation are predicted as A-, B-, C-, E-, G-, and J-type ions using the nomenclature defined in reference [21]. Figure 2a demonstrates that all of A-, B-, C-, E-, G-, and J-type ions predicted in reference [21] were observed for this example and “G+2” ions (mentioned in reference [21]) were observed. The structure of “G+2” ions and their fragmentation pathway are not clear, but “G+2” ions were also observed in the product ion spectrum of the of triolein standard (shown in Figure 5b) at a relatively lower intensity than that of the G-type ions. Figure 2b also shows that signals resulting from charge-remote fragmentation were detected in the high mass range above *m*/*z* 650. When the fragment ion at each bonding position is defined as in Figure 1, the peaks can be assigned as shown in Figure 2b. The intensities of fragment ions corresponding to unsaturated bonding positions, such as L∆9, L∆12, and O∆9, are relatively weak or are not observed, resulting in a peak pattern that reflects the structure of 3 fatty acids.

In the analysis of triacylglycerols in the olive oil sample, particular attention was focused on the G- and J-type ions. These ions have the structure where two molecules of fatty acid are eliminated from the precursor ion [21]. These ions help determine the numbers of carbon chains and unsaturated bonds in each fatty acid. In the G-type ion, fatty acids remain at *sn*-*1*/*sn*-*3*, while the J-type ion, where a fatty acid remains at *sn*-*2*, has one less CH_2_ at the end. This makes it possible to estimate the bonding positions of three fatty acids because fatty acids having an odd acyl carbon number rarely exist in the natural world.

**Figure 3:**
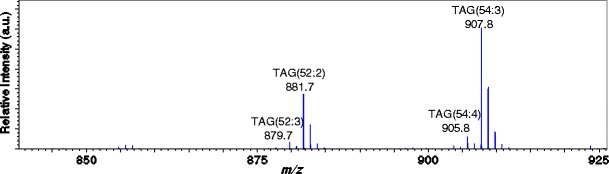
Mass spectrum of olive oil sample showing sodiated TAGs

**Figure 4:**
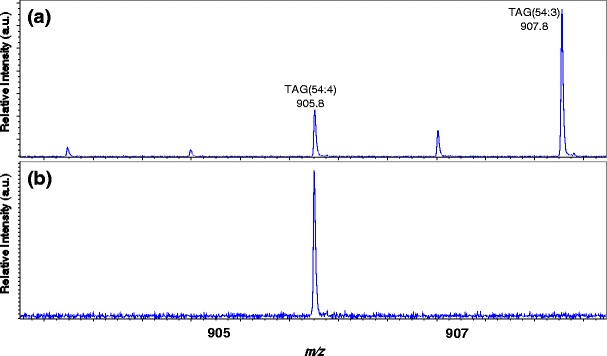
Precursor-ion selection for ions at *m*/*z* 905.8 (**a**) before selection, (**b**) after selection

Figure 3 shows the mass spectrum of the olive oil. Sodiated triacylglycerols [M + Na]^+^ were observed for this sample that included TAG (52:3) (*m*/*z* 879.7), TAG (52:2) (*m*/*z* 881.7), TAG (54:4) (*m*/*z* 905.8), and TAG (54:3) (*m*/*z* 907.8). The monoisotopic ions of these four TAGs were selected as the precursor ions, and their product-ion mass spectra were acquired. Figure 4 shows the spectra of ions at *m*/*z* 905.8 acquired before and after the precursor ion selection. The figure demonstrates that only the ions at *m*/*z* 905.8 were selected, completely eliminating ions at other mass values.

**Figure 5:**
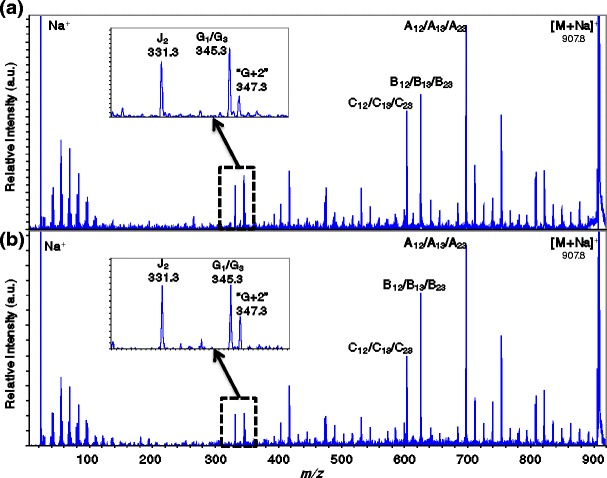
Comparison of product-ion mass spectra for the precursor at *m*/*z* 907.8 with that of sodiated triolein standard, (**a**) the ions at *m*/*z* 907.8 from olive oil, (**b**) triolein standard

Figure 5 shows comparison between the product-ion mass spectra of TAG (54:3) [M + Na]^+^ at *m*/*z* 907.8 from olive oil and from triolein standard. Given that olive oil is rich in oleic acid, the ion at *m*/*z* 907.8 is expected to contain three oleic acids (18:1). Both of the product-ion mass spectra show a J_2_-type ion at *m*/*z* 331.3, indicating that an oleic acid is bonded at the *sn*-*2* position, and a G-type ion at *m*/*z* 345.3, indicating that an oleic acid is bonded at the *sn*-*1*/*sn*-*3* positions. The spectra show only one peak that is considered an A-, B-, and C-type ion, suggesting that TAG (54:3) is trioleoylglycerol, which contains three oleic acid molecules. The signals in high mass region resulting from charge-remote fragmentation were identical between the sample and standard, and the spectral patterns were consistent with structure of oleic acid.

**Figure 6:**
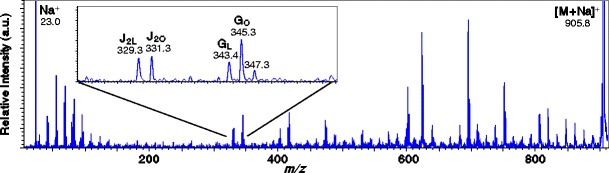
Product-ion mass spectrum for the precursor at *m*/*z* 905.8

Next, the ion at *m*/*z* 905.8 was selected as the precursor ion. The *m*/*z* value of this ion suggests that it is a monoisotopic [M + Na]^+^ ion of TAG (54:4). Figure 6 shows the product-ion mass spectrum. It is expected that this triacylglycerol is also composed of two (18:1) oleic acids and one (18:2) linoleic acid, given that the major component of olive oil is oleic acid. The product-ion mass spectrum shows fragment ions assigned as G-type ions, at *m*/*z* 343.4 and *m*/*z* 345.3. If the ion at *m*/*z* 343.4 is a G-type ion, the fatty acid molecule at *sn*-*1*/*sn*-*3* is linoleic acid, and if the ion at *m*/*z* 345.4 is a G-type ion, the fatty acid molecules at *sn*-*1*/*sn*-*3* are oleic acid. The ion at *m*/*z* 347.3 is assigned as a “G+2” ion because in the product-ion mass spectrum “G+2” ions were observed at lower intensity than G-type ions as discussed above, and the intensity of the ion at *m*/*z* 347.3 is relatively lower than that of the ion at *m*/*z* 345.3. This is consistent with the assignment of G+2 ions by Cheng et al. in reference [21]. Since the G-type ion suggests that both oleic acid and linoleic acid are bonded, the remaining fatty acid is (18:1) oleic acid. Next, the product-ion spectrum shows J-type ions: a J-type ion containing oleic acid (J_2O_) and a J-type ion containing linoleic acid (J_2L_) at *m*/*z* 331.3 and *m*/*z* 329.3, respectively. In the high-mass region, the signals resulting from charge-remote fragmentation were consistent with the structures of oleic acid and linoleic acid. This demonstrates that *m*/*z* 905.8 is triacylglycerol that contains two molecules of oleic acid and one molecule of linoleic acid and is a mixture of the structural isomers 1,3-dioleoyl-2-linoleoyl-glycerol and 1,2-dioleoyl-3-linoleoyl-glycerol. Table 1 summarizes the structures of triacylglycerols determined for the olive oil samples from the product-ion mass spectra. The ions associated with the peak at *m*/*z* 879.7 are a mixture of the structural isomers 1-palmitoyl-2-oleoyl-3-linoleoyl-glycerol and 1-palmitoyl-2-linoleoyl-3-oleoyl-glycerol.

**Table 1:** Summary of TAGs Found in the Olive Oil Sample

*m*/*z*	Acyl carbon number and number of double bond	Composition of each fatty acid	
879.7	52:3	(16:0,18:1,18:2)	(16:0,18:2,18:1)
881.7	52:2	(16:0,18:1,18:1)	
905.8	54:4	(18:1,18:1,18:2)	(18:1,18:2,18:1)
907.8	54:3	(18:1,18:1,18:1)	

## Conclusion

Monoisotopic precursor selection was demonstrated for TOF/TOF analysis of a standard TAG and TAGs in an olive oil sample. This selectivity made it possible to use charge-remote fragmentation to determine the complete structure (except stereochemistry) for all of the TAGs, including structural isomers, present in the sample. Multiple structural isomers in the precursor ion were identified through the observation of G- and J-type ions. These results demonstrate that the MALDI-TOF-TOF system with high precursor ion selectivity can fully analyze the structure of triacylglycerols without prior chromatographic separation, and that the method is effective for the analysis of complex fat composites in food.
